# Taking Down the Walls to the Treatment of Aortic Stenosis∗

**DOI:** 10.1016/j.jacadv.2023.100432

**Published:** 2023-07-28

**Authors:** Sandra B. Lauck, Britt Borregaard

**Affiliations:** a St. Paul’s Hospital, University of British Columbia, Vancouver, Canada; b Odense University Hospital, University of Southern Denmark, Odense, Denmark

**Keywords:** aortic stenosis, racial disparities, surgical aortic valve replacement, transcatheter aortic valve implantation

**Figure:**
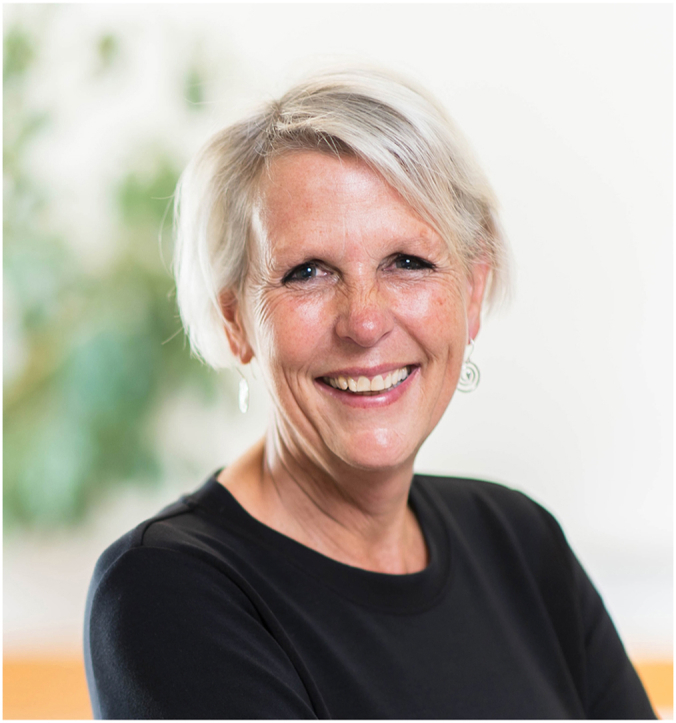

The gap between the imperative to improve health equity and progress made across regions continues to exist and is possibly widening. As clinicians, policy-makers, researchers, and other partners engaged in measuring quality, promoting access, and driving innovation, we have failed to ensure that every person has the opportunity to attain their full health potential, to remove the barriers to high quality and timely care, and to provide equitable access to health innovations regardless of social position or other circumstances. This is apparent across a large body of evidence and highlights the pressing need to do better.

In this issue of *JACC: Advances*, Sevilla-Cazes et al^1^ add to this evidence and offer unique and important insights on the impact of residential racial segregation in the United States on access to care for the treatment of aortic stenosis and to treatment options. Using granular county-level administrative data, they determined that race mattered in the treatment of aortic stenosis: Black Medicare fee-for-service beneficiaries with aortic stenosis living in high-segregation locations were 49% less likely to be diagnosed and 65% less likely to be offered transcatheter aortic valve implantation (TAVI) as a treatment option, in sharp contrast to Black Americans residing in low-segregation counties or White Americans, regardless of residence. In their thoughtful discussion, the investigators draw a compelling line between the impact of systemic racism embodied in racial residential segregation and the root causes of health disparities among people who share a disease but are of different race. They highlight how systemic racism is woven into health services, intersects with patient, system, and disease-related factors, and culminates in disparities that impact outcomes, experiences, and costs.

These findings further affirm the lack of substantial progress in achieving equity in the 20 years.^2^ In 2001, the Institute of Medicine (now the National Academy of Medicine) cited equity as one of the 6 aims for improvement in the report “Crossing the Quality Chasm: A New Health System for the 21st Century.” The subsequent report “Unequal Treatment—Confronting Racial and Ethnic Disparities in Health Care” provided recommendations related to increasing awareness, using policy levers, promoting the use of guidelines, educating patients, equipping clinicians with cross-cultural competencies, and promoting research to identify sources of disparities and develop interventions.^3^ To help realize the unachieved promise of equitable quality improvement, health equity is increasingly recognized as equally important as improved patient experience, better outcomes, lower costs, and clinician well-being—the key elements of the Institute of Healthcare Improvement “Quadruple Aim.” The integration of the principle of health equity in the further revised “Quintuple Aim” reflects the increased awareness that social determinants of health drive 70% of health care outcomes and that efforts to improve cardiovascular care will be futile in the absence of meaningfully driving initiatives to remove systemic barriers.^4^

Our shared task of addressing the complex web of factors that drive inequitable access to care for people living with the debilitating impact of aortic stenosis is daunting. Sevilla-Cazes et al^1^ highlight how the use of intersectionality offers a valuable analytical tool that reflects a strong commitment to ethical values, social justice, and the pursuit of social and health equity. Intersectionality broadens the discussion on the influence of social determinants of health to a recognition of interlocking multiple systems of power. In the context of evaluation and research, it enables a more informed examination of the roots of observed differences in the health of individuals, people and groups, and the development of upstream solutions to redress social and health injustices to achieve improved outcomes and health services.^5^^,^^6^

Looking upstream on the journey of care of people with aortic stenosis, we know that heart valve disease remains under-recognized, underdiagnosed, undertreated, and treated too late across international regions.^7^ These challenges further compound the known barriers to care of marginalized groups, as illustrated by the impact of racialized neighborhoods and other determinants. The Health Policy Partnership and the Global Heart Hub recently produced an important report: *“Heart Valve Disease: Working Together to Create a Better Patient Journey”* to advocate for effective strategies to address the many gaps in patients’ pathway, and urge coalitions of patients and families, clinicians, and policy-makers to advocate for concrete actions to shift the culture of the management heart valve disease.^8^ In partnership with patients, strategic partners have established recommendations to strengthen disease awareness, diagnosis, and access to echocardiography, seamless referral, work-up and monitoring, timely treatment and comprehensive follow-up.^9^ The challenge now lies on incorporating equity as a guiding principle to help dismantle the barriers that many people face to access this journey in health policy initiatives.

These efforts can be accelerated by empowering patients with the information required to seek treatment early and participate in making a high-quality treatment decision. The significant advances made in raising people’s awareness and urgency to act when experiencing chest pain can serve as an inspiring model to translate these successes to valvular heart disease and invest in efforts to support community outreach and equitable disease surveillance. As highlighted by Sevilla-Cazes et al,^1^ this will require addressing systemic barriers, such as uneven insurance coverage, distrust in the medical system, inconsistent culturally competent care, and the competing pressures of poverty. Applying an equity lens to the promotion of shared decision-making—an exchange of information that invites patients and families to communicate what matters most to them in their treatment decision^10^—may help address the unsettling finding that Black Americans living in high segregation areas were more likely to have surgery than TAVI, in spite of having similar outcomes when TAVI was performed. This finding reminds us that innovation in health care is not neutral—new technology and health services can inadvertently maintain, worsen, or introduce inequities.^11^ Efforts are needed to ensure that equitable access to guideline-driven care in the current era of demonstrated excellent outcomes and early quality of life benefits of TAVI ought to be available to all.

Closing the gap between *what we know*—there are unjust differences in access, use, quality, and outcomes of care in the identification and treatment of aortic stenosis between people based on multiple intersectional factors, including residence—and *what we do*, as clinicians, researchers, policy-makers, funders, and advocacy groups is essential to move evidence “off the shelves, and onto the streets.”^12^ To this end, reframing knowledge translation efforts—both through the activities undertaken to move evidence into practice, and the scientific study of the methods to promote the uptake of research in clinical, organizational, or policy contexts—to address inequities in health care delivery can help move the focus from awareness to action. We can leverage the common goals of health care inequity research and knowledge translation to pursue an agenda that emphasizes the importance of contextual factors and the impact of systemic racism, and values multilevel approaches to address the constellation of determinants that drive inequity across populations and health services.^13^ To achieve this vision of quality improvement across complex cardiovascular health services, we need to center efforts to dismantle factors that perpetuate inequities within systems that have not been intentionally designed to eliminate racial or other intersecting differences in access and outcomes.^14^

The provocative findings presented by Sevilla-Cazes et al^1^ compel us to drive the significant efforts needed to move from awareness to action. The roadmap to advance cardiovascular health can only succeed if it includes creating a culture of equity, identifying disparities and their root causes, and designing, implementing, and measuring interventions that will result in taking down the walls to the treatment of aortic stenosis.

## Funding support and author disclosures

Dr Lauck is supported by the St. Paul's Hospital Professorship in Cardiovascular Nursing at the University of British Columbia; and has provided consulting services for Edwards and Medtronic. Dr Borregaard has reported that he has no relationships relevant to the contents of this paper to disclose.
